# Inactivation, Aggregation and Conformational Changes of Polyphenol Oxidase from Quince (*Cydonia oblonga* Miller) Juice Subjected to Thermal and High-Pressure Carbon Dioxide Treatment

**DOI:** 10.3390/molecules23071743

**Published:** 2018-07-17

**Authors:** Aamir Iqbal, Ayesha Murtaza, Zafarullah Muhammad, Abdeen E. Elkhedir, Mingfang Tao, Xiaoyun Xu

**Affiliations:** 1 Key Laboratory of Environment Correlative Dietology, Huazhong Agricultural University, Ministry of Education, Wuhan 430070, China; aamirraoiqbal@webmail.hzau.edu.cn (A.I.); ayesha_murtaza2003@hotmail.com (A.M.); zafmuhammad@mail.hzau.edu.cn (Z.M.); abdeensiddig@yahoo.com (A.E.E.); mingfang432@outlook.com (M.T.); 2 College of Food Science and Technology, Huazhong Agricultural University, No. 1, Shizishan Road, Wuhan 430070, China

**Keywords:** polyphenol oxidase, high pressure carbon dioxide, aggregation, λmax, conformation

## Abstract

Polyphenol oxidase (PPO) causes the browning reaction in fruits and vegetables and deteriorates the quality. Thermal treatment for enzyme inactivation may result in defects as opposed to high pressure CO_2_ (HPCD) processing. In this study, the changes in activity, dissociation, aggregation and conformation of purified PPO from thermal and HPCD treated juice were investigated. HPCD exhibited inactivation of PPO at 55–65 °C whereas thermal processing alone at the same temperature resulted in PPO still showing activity. Under thermal treatment at 25 and 65 °C, the browning degree was higher (0.39 and 0.24) than for HPCD-treated juice (0.23 and 0.12). Fluorescence and circular dichroism spectral results indicated that HPCD induced large decreases in intensities, revealing a rearrangement of the secondary structure and destruction of the native configuration of the PPO molecule. The particle size distribution (PSD) pattern revealed structural modification leading to initial dissociation and subsequent aggregation of PPO after HPCD treatment. Polyacrylamide gel electrophoresis (PAGE) analysis exhibited that molecular size of protein was 40 kDa. In conclusion, the HPCD method was found to be more effective than thermal treatment to inactivate PPO. Structural modifications provided better insights into the phenomena of activation and inactivation of PPO.

## 1. Introduction

Quince (*Cydonia oblonga* Miller) is the fruit of the Maloideae, a sub-family of the Rosaceae family [1]. Commercially significant fruits including apples and pears also belong to this family [2]. Quince is considered a functional fruit due to its medicinal and nutritional values and is mostly used in the production of jam, marmalades, jelly, liquor and cakes. Quince fruit has gained much attention in the last decade due to its high antioxidant capacity resulting from phytochemicals including phenolic compounds such as hydroxycinnamic acid and proanthocyanidins [1,2,3]. Storage conditions and further processing cause enzymatic browning of fruit, leading to physical and chemical damages resulting in undesirable quality depletion [4]. The browning phenomenon is mainly due to the high activity of enzymes from the oxidoreductase group such as polyphenoloxidase (PPO, EC 1.14.18.1) and peroxidase (POD, EC 1.11.1.7) [5]. Within the oxidoreductase group, PPO is mainly responsible for the formation of brown pigment by oxidation of polyphenol. The PPO is considered as a copper protein. The active site of the PPO enzyme contains two copper ions, which are surrounded by 6 histidine residues and a single cysteine residue. It also serves as structural support for enzyme molecule, thereby affecting the activity of protein. Copper ions present in the active site of PPO are responsible for oxidation-reduction processes that lead to the transition of active sites among met-, oxy-, and deoxy-forms. Thus, copper plays a significant role in the structure and activity of the PPO enzyme [6]. The PPO is an enzyme comprised of two subunits (H and L). The subunit H has a binuclear copper-binding site in the deoxy-state while L subunits coordinate each copper ion to catalyze two different reactions involving molecular oxygen; oxidation of monophenols (*O*-hydroxylation) to *O*-diphenols and *O*-diphenols to *O*-quinones, which are further responsible for the polymerization of brown pigments [7,8].

Thermal treatment has been the traditional technology for the inactivation of enzymes. Heat also causes detrimental changes in the food properties, i.e., loss of flavor, color, nutrients and texture. Various innovative technologies including high pressure carbon dioxide (HPCD), high-hydrostatic pressure (HHP), ultra-sonication, and irradiation have been used to reduce the detrimental changes in various fruit and vegetables caused by thermal technology [4,9]. HPCD is considered an innovative and emerging non-thermal technology, which is an alternate to traditional heating or high-pressure processing of fruit juices and is deemed comparatively safe for those compounds which are heat-liable [10,11]. The literature suggests that HPCD is efficient in the inactivation of PPO [12,13]. HPCD utilizes CO_2_ properties which can inactivate enzymes by pH lowering, cell disruption, cell membrane modification and cellular component extraction [14]. The inactivation of PPO in processed fruits and vegetables is the main index of quality. Numerous studies on the PPO enzyme from several sources, i.e., apple, banana, pear, carrot, celery and loquat, have reported enzyme characterization, thereby controlling their actions [15,16].

The enzymatic inactivation of PPO, quality parameters and structural analysis of purified PPO from quince juice treated with thermal and non-thermal technology (HPCD) are addressed in the present paper. To our knowledge, no work has been done on fruit quality attributes, enzymatic inactivation and structural modification of PPO purified from juice after thermal and HPCD treatments. Quince juice was exposed to varying heat and HPCD treatments of varying temperatures and pressure. Structural analysis was conducted to explore the mechanism of thermal and HPCD technology on the aggregation and deformation of the protein after purification with the activity of the polyphenol oxidase enzyme.

## 2. Materials and Methods

### 2.1. Extraction of Quince Juice

Fresh quince (*Cydonia oblonga* Miller) at optimum maturity was bought from a native market in Xinjiang, China. Fresh quince was washed, peeled and cut into slices. Slices were crushed with a juice extractor with 0.1% (*w*/*w*) ascorbic acid added to inhibit undesirable enzymatic browning. The juice was filtered through a cheese cloth to remove coarse particles and then centrifuged at 4000 rpm for 5 min. The resulting juice was subjected to thermal and HPCD treatments.

### 2.2. Thermal and HPCD Treatments

#### 2.2.1. Untreated Quince Juice

For each experiment, 30 mL of freshly squeezed juice without heat or HPCD treatment was used as the control.

#### 2.2.2. Thermal Processing of Juice

Thermal treatment was carried out in a water bath at varying temperatures (25–65 °C) with a 10 °C increase for 20 min. Quince juice (30 mL) was poured into 50 mL plastic tubes. The treatment time was started when the desired temperature of the quince juice as measured by digital thermometer (Wuhan, China) was reached. After the thermal treatment, the samples were immediately placed in a refrigerator for further analysis. Enzyme activity and other quality parameters were then determined.

#### 2.2.3. HPCD Processing of Juice

HPCD treatment followed the method of Liao et al. [17] in which 99.5% pure CO_2_ obtained from Wuhan Co. (Wuhan, China) was introduced into the pressure vessel. Quince juice (30 mL) was transferred into a 50 mL sterile plastic bottle. For each experiment, the HPCD pressure vessel was sanitized with ethanol and preheated to the experimental temperature before juice samples were placed inside. After the desired temperatures of 25, 35, 45, 55 and 65 °C were reached, the chamber vessel was pressurized to 20 MPa with a plunger pump. The quince juice in the chamber vessel was held at constant temperature and pressure for 20 min. The pressure ramp used was 20 MPa/30 s, and at the end of the treatment, the chamber vessel was gradually depressurized for 1.5–2 min. Pressurization and depressurization time was not included in treatment time. Following the HPCD treatment, quality parameters of the quince juices were evaluated.

### 2.3. Quality Attributes of Quince Juice

#### 2.3.1. Colorimetric Measurement

Colorimetric measurement of quince juice was performed at ambient temperature with a chromometer (CR-410; Konica Minolta Corporation, Osaka, Japan). The colorimetric values of L*, a*, and b* were measured, where L* indicated lightness, a* indicated redness, and b* indicated yellowness.

#### 2.3.2. Browning Determination

The browning degree of quince juice was evaluated using a Multiskan spectrophotometer (Thermo Scientific, Waltham, MA, USA) [14]. The sample solution was centrifuged (10,000 rpm) for 20 min, and the BD was measured after adding 100 μL of sample to the enzyme-linked immunosorbent assay (ELISA) plate and tested immediately at λ = 420 nm by using a simple kinetic method.

#### 2.3.3. Physico-Chemical Analysis

The juice pH was determined using a digital Thermo pH meter (Thermo Fisher, Waltham, MA, USA). Total soluble solid (TSS) contents were measured by a digital Abbe refraction meter (Shanghai Precision and Scientific Instrument Co., Shanghai, China).

### 2.4. PPO Extraction and Purification

Quince juice was collected after treatments (thermal and HPCD) and PPO extraction and purification methods followed our former study [9] in which 25% (NH4)_2_SO_4_ saturation was used to fractionate the juice to remove impurities, followed by 80% saturated solution for 1 h for protein precipitation. Juice was then subjected to centrifugation at 15,000 rpm for 20 min. Protein precipitates were resuspended in 0.5 mol/L Tris-HCl buffer (pH 7.0) and were dialyzed against Tris-HCl buffer for 24 h and concentrated by ultra-filtration and purified by using DEAE sepharose fast flow columns (1.0 × 10 cm). Fractions containing higher PPO activity were selected for further analysis. The fractions with the highest PPO activity were concentrated and stored for further analysis.

### 2.5. PPO Activity

The relative activity of crude and purified enzyme was measured by following the method presented by Liu et al. [11] with slight modifications Crude enzyme was made by adding 0.1% polyvinylpolypyrrolidone (PVPP) and (0.1%) Triton X-100 to 2 mL quince juice. After a storage time of 1 h at 4 °C, the juice was centrifuged at 10,000 rpm for 20 min. This crude enzyme was used for PPO activity. An aliquot of 50 μL supernatant was mixed with 0.1 M catechol substrate solution (200 μL) and with 0.05 M phosphate buffer (pH 7.2). Absorbance of the solution was determined at a wavelength of 420 nm at 30 s intervals for 3 min (30 °C incubation) in a spectrophotometer (Multiskan FC-Thermo Scientific, Waltham, MA, USA). The PPO specific activity (Abs/min) was taken as the first linear part of the slope from the reaction curve. The relative activity (RA) was determined using the following equation:

$$\;\mathrm{Relative}\;\mathrm{activity}=\;-\frac{\mathrm{Activity}\;\mathrm{of}\;\mathrm{juice}\;\mathrm{after}\;\mathrm{thermal}\;\mathrm{or}\;\mathrm{HPCD}\;\mathrm{treatment}}{\mathrm{Activity}\;\mathrm{of}\;\mathrm{untreated}\;\mathrm{juice}}\;\times \;100\;$$

### 2.6. Protein Determination

Protein contents were analyzed according to the Bradford method at a wavelength of 595 nm. Bovine serum albumin (BSA) was used as the standard for protein [18].

### 2.7. Electrophoretic Analysis of Thermal and HPCD-Treated PPO Enzymes

Sodium dodecyl sulphate (SDS) and non-denaturing polyacrylamide gel electrophoresis (Native-PAGE) was used to identified the purification process by using the Mini-proten 4 cell system (Bio-Rad, Hercules, CA, USA) to determine the molecular mass of purified PPO [15]. Gels were used to evaluate the purity and molecular weight of purified PPO protein.

### 2.8. Structural Analysis of Untreated and Treated (Thermal and HPCD) PPO

#### 2.8.1. Circular Dichroism Spectral Measurement

Circular dichroism analysis was conducted according to earlier studies [19,20] with a JASCO spectropolarimeter (JASCO, Tokyo, Japan). CD measurements were recorded by using optical path-length 0.009 cm quartz: 0.2 mg/mL protein sample was dissolved in 50 mmol/L Tris-HCl buffer (pH 7.0), using Tris buffer as a blank sample. CD parameters were observed in the ultraviolet range of 190–240 nm for 2 s and 1 nm bandwidth at 100 nm/min. The CD data were expressed as mdeg (molar extinction coefficient difference) and the protein secondary structure was estimated from Yang’s equation.

#### 2.8.2. Fluorescence Spectral Measurement

Intrinsic fluorescence spectral analysis was conducted using a fluorescence spectrophotometer (F-4600, Hitachi, Tokyo, Japan). Protein samples (0.2 mg/mL) were subjected to spectrophotometer analysis to determine the maximum excitation wavelength. Protein samples were scanned for emission spectra at λem 300–400 nm of protein. The emission and excitation slit width (em and ex) were adjusted at 5 nm. The speed was set as 240 nm/min, and a 0.1 s response time was used [15].

#### 2.8.3. PSD Measurement

Particle size distribution (PSD) was measured by a Zetasizer Nano-ZS device (Malvern Co., Malvern, UK) according to the description of Hu et al. [21]. The purified protein samples from HPCD and thermal treated juice were adjusted to 0.3 mg/mL with phosphate buffer (50 mM, pH 7.0) for determination of protein PSD. A wavelength laser of 532 nm of light scattering with a 15° reflection angle was applied to the sample solution. The size measurement results containing PSD analysis were taken as the mean from four readings.

### 2.9. Statistical Analysis

Data were analysed by ANOVA (analysis of variance), and mean comparisons were made by Tukey’s test. The mean was taken at a significantly different 95% confidence interval (*p* ≤ 0.05). The graphs were constructed using Origin 9.0 software (Origin Lab, Northampton, MA, USA).

## 3. Results and Discussion

### 3.1. Changes in Color, pH and TSS of Quince Juice

**Figure 1:**
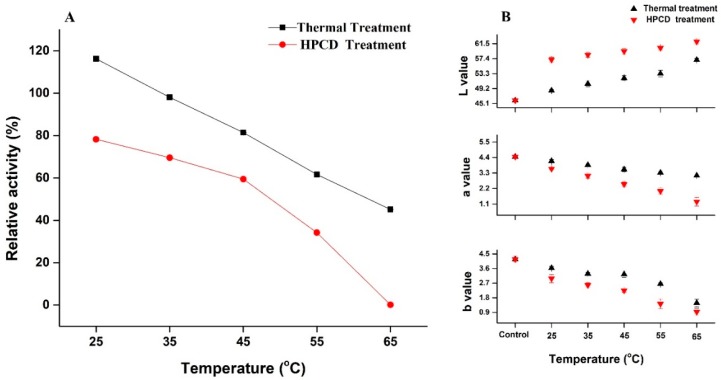
(**A**) Relative activity of PPO; (**B**) Color parameters (L*, a* and b*) of quince juice treated with thermal and HPCD treatment at different temperatures.

The color parameters of juice after HPCD and thermal treatment (25–65 °C) are shown in Figure 1B. The redness and yellowness indicated by a* and b* values of the quince juice after thermal treatment were less than the control, and gradually decreased with increasing temperature. The color value (a* and b*) of HPCD-treated quince juice was significantly lower compared to the thermal-treated juice. An increase in brightness indicated by the L* value was observed with a temperature increase during both HPCD and thermal treatments. A similar finding was reported by Yu et al. [22], i.e., in banana juice, browning development was slower in HPCD juice as compared to thermally treated and control juice. Color changes in quince juice could result from browning caused by PPO, which leads to the deterioration of juice quality.

The influence of HPCD and thermal treatment on pH and TSS of quince juice is presented in Table 1. The pH value of untreated juice was 4.74, but after HPCD treatment, decreased to 4.23, 4.20 and 4.11 at 25, 45 and 65 °C respectively. Thermally treated juice showed only a slight decrease in pH as compared to the control. The pH decline after HPCD treatment could be attributed to CO_2_ dissolving in the juice, which further distributes carbonate, H+ ions and bicarbonates, thus increasing the acidity and lowering the pH value. This finding was in accordance with studies of carrot, watermelon and apple juice in which pH was reduced significantly after HPCD treatment [16,23]. pH is important in that it can be associated with enzyme inactivation. At lower pH, CO_2_ may more easily interact with the enzyme-bound arginine, resulting in a protein complex which can cause the loss of enzymatic activity [24]. According to structural analysis, it has been proved that basic amino acids like arginine, lysine and histidine are those most commonly found in CO_2_-protein binding sites. HPCD treatment could cause the formation of complex covalent carbamates with free amino acid groups on the enzyme surface that were potentially responsible for reducing enzyme activity [10,25]. Untreated quince juice showed a TSS value of 11.55 °Brix. After HPCD and thermal treatment, TSS values did not alter significantly (Table 1). During storage, TSS also did not change significantly, indicating that the processing treatments did not impact soluble carbohydrates such as glucose and sucrose that are considered the major contributors to TSS.

### 3.2. Activation & Inactivation of Crude PPO Enzyme by Thermal and HPCD Treatments

The RA of quince crude PPO during thermal and HPCD (20 MPa) treatments at 25 to 65 °C for 20 min is shown in Figure 1A. PPO after thermal treatment showed the highest RA (116.22% and 98.09%) at 25 and 35 °C. respectively. RA gradually decreased to 81.46% at 45 °C and showed a more rapid decrease to 61.64% and 45.14% at 55 and at 65 °C respectively. PPO is present in numerous (mature, immature, latent and active forms) isoforms, and its increased activity at 25 °C (TP) may be due to the presence of latent PPO [15,26]. In comparison, the results of HPCD treated quince juice showed that the RA decreased to 78.26%, 69.55%, 59.45% and 34.18% with increased temperatures of 25, 35, 45 and 55 °C respectively. Nearly complete inactivation occurred at 65 °C (Figure 1A). HPCD-treated juice significantly lowered the PPO activity as compared to thermal-treated juice at same temperature. Research reports suggest that PPO inactivation of juice under HPCD treatment was improved with increased pressure, temperature and treatment time [22,27]. Enzymatic inactivation under HPCD conditions may be due to changes in the secondary structure of PPO, which might be the result of pH lowering after CO_2_ dissolved in solution. Li et al. also reported that enzymatic activity of apple PPO under HPCD treatment showed biphasic changes of initial activation and then sudden inactivation at 35–55 °C for 15 min [20]. Similar results were found in banana pulp, where PPO activity under HPCD gradually decreased from 40.7% to 11.6% with treatment at 45–60 °C for 30 min at 20 MPa [22]. The results of relative activity of PPO after purification are shown in Table 2. Compared to crude PPO activity (116.22%), the lower activity (46%) was observed for purified PPO using 25 °C under thermal treatment. The lowest relative activity (7.40%) was observed for purified PPO subjected to 65 °C under HPCD treatment. The activity of the crude PPO enzyme was found to be higher than purified enzyme in our study. This might be due to the reason of ammonium sulphate saturation during enzyme extraction, which decreases the level of PPO activity. Dialysis of protein precipitate during purification also causes loss of copper ions, which also decrease PPO activity [28]. It is generally believed that loss of PPO activity may be due to PPO forming some inactive phenols [29] and also because of a good correlation between the α-helix content and residual activity under HPCD treatment. The irreversible decomposition of α-helix occurred after HPCD. Total inactivation of PPO leads to structural modification and denaturation of the protein. These phenomena showed that PPO activity can be irreversibly inactivated by HPCD [24]. It is generally believed that browning is due to PPO-mediated oxidation of phenols. In our study, it was determined that inactivation of PPO in quince juice occurred under HPCD treatment, suggesting that HPCD performs better than thermal treatment alone.

### 3.3. Effect of Thermal and HPCD Processing on Browning Degree of Quince Juice

**Figure 2:**
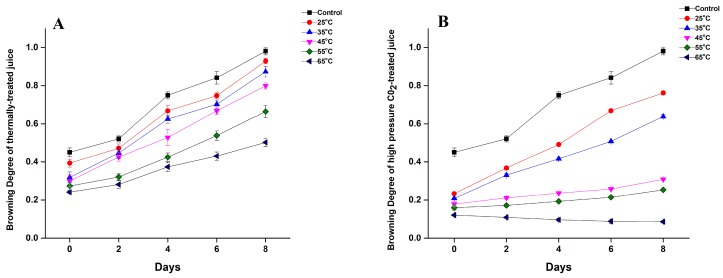
Browning degree during storage at 4 °C (**A**) Thermal treatment; (**B**) HPCD treatment of quince juice at different temperatures.

The BD of quince juice during storage at 4 °C after HPCD and thermal treatment is shown in Figure 2. Untreated juice initially had a browning degree of 0.45 but increased to 0.98 after 8 days of storage. HPCD reduced the BD value compared to thermally treated juice (Figure 2A). BD decreased with increasing temperature and with days of storage. The BD of thermally treated quince juice at 25 °C was 0.39 and it decreased to 0.27 and 0.24 as the temperature increased to 55 and 65 °C (Figure 2A). In addition, HPCD treatment was found to be more effective in controlling browning, as HPCD-treated quince juice on day 1 showed BD values of 0.23 and 0.12 at 25 and 65 °C, respectively, while thermal-treated juice exhibited values of 0.39 and 0.24 (Figure 2B).

Similar results for the reduction of BD were reported for HPCD-treated apple and water melon juice in comparison to thermally treated juice [27,30]. The BD of carrot juice was also reported to decrease from increased pressure and treatment time of HPCD. Under HPCD treatment at 20 MPa for 15 min (25 °C), the BD of carrot juice significantly decreased to 0.272, whereas untreated juice showed a higher value of 0.779 [31]. BD is closely related to enzyme actions, especially PPO. HPCD treatment can inactivate enzymes more efficiently as compared to thermal treatment for prevention of the browning of quince juice.

### 3.4. Particle Size Distribution of Thermal- and HPCD-Treated PPO

**Figure 3:**
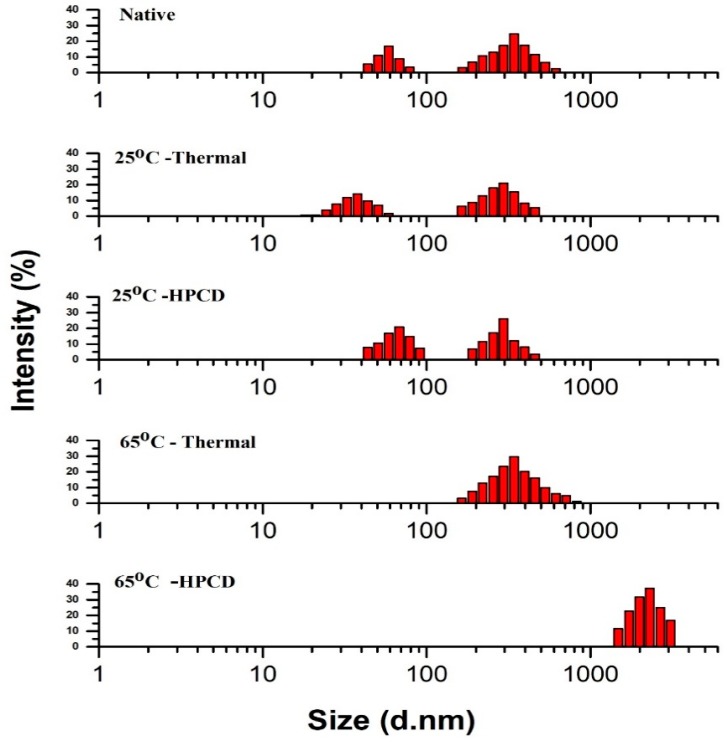
Particle size distributions of native PPO in comparison to thermal- and HPCD-treated PPO.

Thermal and HPCD treatments affected the PSD pattern of purified PPO as shown in Figure 3. The native PPO exhibited a wider span with two Sections (I and II); Section (I) revealed that native protein partially aggregated at 58.8 nm with the fraction containing 16.76%, while mostly PPO aggregates were distributed in section II, which exhibited a particle pattern ranging from 165 to 615 nm with the highest fraction (24.59%) at 342 nm. Peak diameter results suggest that the small sized proteins tend to aggregate to form large accumulates of PPO. For thermally treated PPO at 25 °C, lower intensities with two sections were observed: Section I at 37.8 nm with the fraction occupying 14.09%, and Section (II) with some partially aggregated protein (295 nm) with fractions occupying 20.92%. At 65 °C, the PSD pattern showed one section with a fraction value of 29.6 at 342 nm. HPCD at 25 °C exhibited a higher intensity than the thermal treatment (25 °C) with two sections; Section (I): 68.1 nm with a 20.77% number fraction and Section (II): 26.19% at 295 nm. At 65 °C with HPCD, the span of the particle diameter further increased and PPO was strongly inactivated and denatured.

Several studies have reported that HPCD treatment resulted in aggregation and dissociation of protein. HPCD at high temperature caused dissociation of large aggregates with high particle diameter, indicating small aggregates with small particle diameters [20,32]. It has also been shown that intermediates might exhibit size differences from the native structure, resulting in different properties [23]. High temperature and HPCD treatment induced small particle aggregation and larger particle dissociation [20]. The PSD pattern of purified PPO from quince after HPCD treatment showed dissociation and formation of aggregates. Enhanced interactions among protein molecules resulting from the catalytic site increased the inactivation rate by reduced particle size, resulting in dissociation and disintegration of the protein structure [15,33]. PPO with a small size may associate to form aggregates at high temperature with further aggregation leading to significant accumulation, thereby enhancing the distribution span [11]. High temperature under HPCD treatment enhanced the homogenization. The aggregation induced by HPCD was probably due to a reduction in pH. CO_2_ reacts with water to form carbonic acid, which reduces the environmental pH. Hence, reduced pH caused folding and unfolding of protein chains, which lead to aggregation. Furthermore, carbonic acid may react with copper atoms and the histidine residues present at the catalytic site, destroying the active catalytic centre and causing inactivation of PPO [21].

Additionally, depressurization of CO_2_ after HPCD treatment caused the formation of a gas liquid interface. Nonpolar residues occurred in the gas phase while polar particles oriented to the liquid phase, causing unfolding of protein chains. Thus, unfolded molecules will undergo aggregation of particles. Meanwhile, depressurization causes rapid gas expansion from enzymes. This gas expansion induced an explosive effect and a homogenization effect of aggregates [20,21].

In this study, compared to thermal treatment, the PSD pattern of HPCD-treated purified PPO showed significant variations with broader spans of larger particle diameters resulting in massive changes in PPO conformation. These structural changes including fragmentation, deformation and aggregation affected the active catalytic site of PPO molecules and altered the secondary structure, resulting in PPO activation and inactivation. PSD pattern of HPCD-treated PPO from quince showed marked significant variations with broader spans of larger particle diameters resulting in massive changes in PPO conformation.

### 3.5. Effect of Thermal and HPCD Treatments on the Secondary Structure of PPO

**Figure 4:**
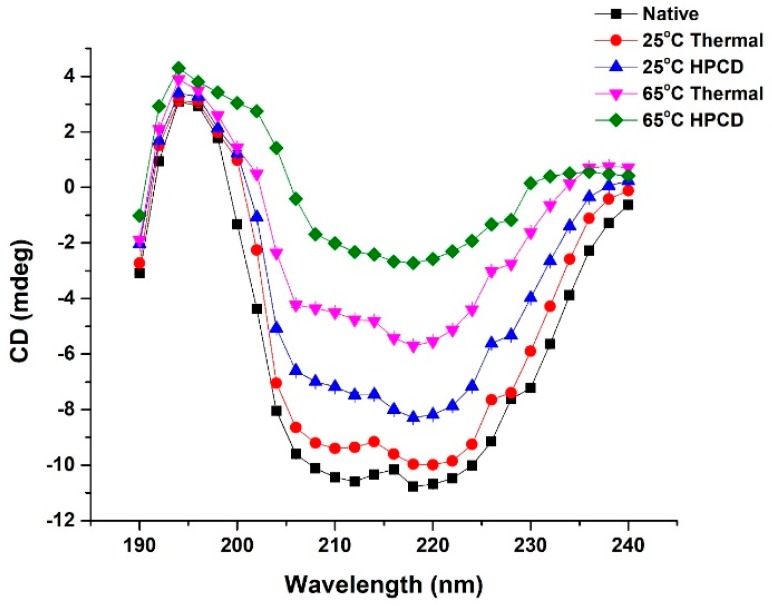
CD spectra of native PPO in comparison to thermal- and HPCD-treated (25 & 65 °C) PPO.

CD spectra are a valuable spectroscopic technique to investigate changes in secondary structure including the α-helix, β sheet and β-turn of PPO protein. The α-helix contents were calculated for negative elliptical peaks of 222 and 208 nm while the 214 nm peak is a characteristic of a β-sheet [15,34,35]. The CD spectra of native PPO in comparison to HPCD and thermally treated PPO are shown in Figure 4. The native purified PPO exhibited a negative peak at 218 nm and a positive peak at 194 nm (Figure 4). The data suggest that native PPO possesses an α-helix arrangement in secondary constellation. The CD spectra of thermally treated PPO at 25 °C showed minor modification from native PPO, indicating no structural changes. However, at 65 °C, considerable changes in PPO secondary structure resulted. Compared to the native PPO, the positive peak value at 194 nm changed slightly at 25 °C but gradually increased as pressure was applied for 20 min. The negative ellipticity increased and the positive peak value decreased by increasing temperature under HPCD, suggesting the loss in the α-helix content and enhanced structure disorder. Moreover, the CD spectra of HPCD-treated PPO protein showed major changes in secondary structure as compared to thermally treated PPO.

The estimation of the secondary structure showed that thermal and HPCD treatments caused a marked reduction in the contents of the α-helix and β-turn and enhanced the contents of the β-sheet and random coil (Table 2). Native PPO consisted of 39.34% α-helix, 17.9% β-sheet, 26.41% β-turn and 16.35% random coil. The secondary structure of thermally treated purified PPO showed little change at 25 °C, but at 65 °C, the α -helix decreased to 38.1% while the β-sheet and random coil increased to 23.40 and 24.58% respectively. Thus, loss of α-helix contents disorganized the native structure of PPO and eventually enhanced the negative ellipticity of CD spectra. Moreover, when pressure was applied in combination with temperature at 65 °C, the α-helix and β-turn content decreased to 24.11% and 18.40% while the contents of the β-sheet and random coil increased to 27.70% and 29.79%, respectively (Table 2). High temperature damaged the native structure of the protein, leading to the reorganization of secondary structure. The β-sheet content of PPO increased after HPCD treatment, facilitating aggregation. This might be due to a sudden release of CO_2_ causing rapid gas expansion, inducing explosive and homogenization impacts on the protein aggregates [20].

A previous study stated that thermal treatment resulted in a reduction of the α-helix content in mushroom PPO, while β-sheet and random coil contents increased. Temperature increase also influenced the structural changes in PPO [35,36]. Our HPCD results are consistent with Li et al. [20] in which a decline in α-helix contents followed by decrease in PPO activity was observed in amyloid-forming aggregates. CD spectra revealed that purified PPO is correlated with PPO activity. According to our results of CD and PPO activity, the increases in β-sheet and β-turn contributed to the greater change in PPO conformation, indicating the collapse of the catalytic center. The HPCD treatment caused changes in the α-helix content, which altered the interaction between the copper centre and the α-helix site, collapsing the active site, and thus resulting in PPO inactivation [24].

HPCD may change hydrogen bonding interactions within PPO, leading to conformational changes of the secondary structure and ultimately resulting in PPO inactivation.

### 3.6. Fluorescence Spectroscopy Analysis

**Figure 5:**
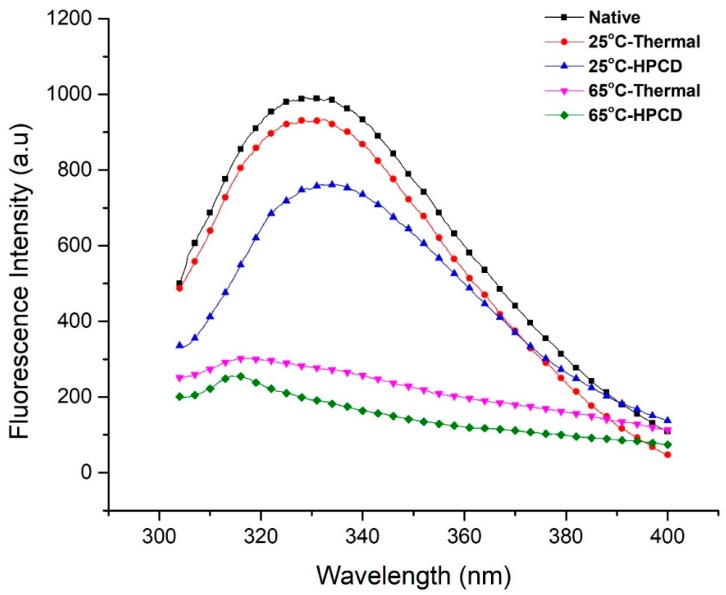
Fluorescence spectra of PPO during thermal treatment at 25 and 65 °C and HPCD treatment at 25 and 65 °C and its correlation analysis during PPO inactivation.

As previously described, variations in enzyme tertiary structure relate to the changes in intrinsic fluorescence emission. The polarity of protein residues including tryptophan and tyrosine provides a valuable technique to characterize protein conformation [15]. Thermal- and HPCD-treated purified PPO was examined by fluorescence spectroscopy (Figure 5). Native PPO displayed the largest emission peaks at 331.2 nm (λmax) with fluorescence intensity of 990 a.u. The data indicate that tryptophan remnants were located in the nonpolar environment [20]. Under thermal treatment (25 °C), λmax was slightly red-shifted to 332.6 nm and its intensity decreased to 933.2 a.u., while the λmax of HPCD-treated PPO at the same temperature increased rapidly to 334.8 nm and displayed 3.6 nm red shifts as compared with native PPO. However, when the temperature increased from 25–65 °C at 20 MPa, the florescence spectrum exhibited blue shifts at λmax 314.2 nm and its intensity decreased from 761.6 to 255.7, indicating the loss of fluorescence intensity of PPO when 20 MPa pressure was applied [29]. Hence, high temperature showed enormous changes with blue shifts and reduced fluorescence intensity in fluorescence spectra, causing inactivation of PPO. A possible explanation is that an increase in temperature resulted in amino acid exposure and unfolding of PPO molecules, causing disruption of the tertiary structure and a subsequent decrease in fluorescence intensity. Previous studies agree with our findings, that thermally treated PPO proteins at high temperature (75 °C) formed aggregates after losing their shape. Ultimately, this resulted in dislocation of the native structure of PPO and brought about unfolding or deformation of the structure [36]. Zhou et al. [34] also suggested that at 70–80 °C, PPO might conglomerate, averting a decrease in fluorescence intensity. PPO protein was denatured and formed aggregates, suggesting the strong influence on inactivation of PPO for an extended storage period. During structural changes in the enzyme, buried fluorophores moved interiorly or were exposed to the outside, which causes changes in fluorescence intensity [15]. Hence, in our study, λmax was red-shifted due to inward movement of fluorophores in the structure of purified PPO which caused the reduction of fluorescence intensity. As a result, intensity reduction can be attributed to inward burying of fluorophores.

**Figure 6:**
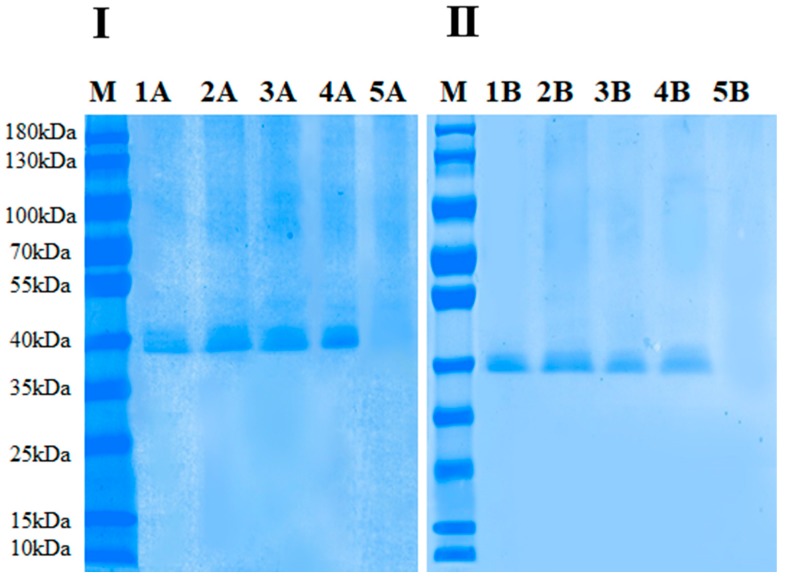
(**I**) SDS-PAGE analysis of the PPO protein: M: Marker, 1A: native PPO, 2A: 25 °C-treated protein (thermal treatment), 3A: 25 °C-treated protein (HPCD treatment), 4A: 65 °C-treated protein (thermal treatment), 5A: 65 °C-treated protein (HPCD treatment); (**II**) Native PAGE analysis of PPO protein: M: Marker, 1B: native PPO, 2B: 25 °C-treated protein (thermal treatment). 3B: 25 °C-treated protein (HPCD treatment), 4B: 65 °C-treated protein (thermal treatment), 5B: 65 °C-treated protein (HPCD treatment).

This present work confirmed other reported research findings in which HPCD-treated protein with high PPO activity showed that fluorescence intensity was significantly reduced at higher temperature, pressure and time [20]. Compared to the native and thermal-treated PPO, HPCD-treated PPO also revealed blue-shifted λmax with large decreases in intensities. These findings may correspond to the establishment of a more polar environment, resulting in higher fluorescence spectrum quenching. SDS-PAGE and Native PAGE results of purified PPO under thermal and HPCD treatments are shown in Figure 6. All proteins showed bands except at 65 °C under HPCD treatment due to complete inactivation of enzyme. Polyacrylamide gel electrophoresis (PAGE) analysis showed that the molecular size of the protein was 40 kDa. Compared to native PPO activity (24.23 ± 0.76), the lowest relative activity (07.40 ± 1.33) was observed for purified PPO using HPCD treatment (Table 2). This finding proves that inactivation of the enzyme under thermal and HPCD treatment resulted in a blue shift of the fluorescence spectra. Thereby changes in fluorescence spectra were closely related to the PSD and activity of PPO (Figure 5). HPCD was more effective in inactivation of PPO as compared to thermal processing, resulting from a high disruption of the PPO tertiary structure by exposing PPO to a more polar environment.

## 4. Conclusions

In this study, HPCD treatment was more effective in lowering the PPO activity and structural modification of quince PPO in comparison to thermal treatment. HPCD treatment resulted in inactivation and conformational changes of quince PPO. The relative activity of PPO and BD of juice decreased with an increase in temperature with HPCD. The activity of the crude PPO enzyme was found to be higher than purified enzyme due to loss of copper ions during purification of the enzyme. The PSD pattern after HPCD treatment revealed that structural modification of PPO occurred, leading to initial dissociation and subsequent aggregation. Intrinsic fluorescence spectroscopy indicated that HPCD induced blue shifts in λ_max_ with large decreases in intensities as compared to thermal processing resulting from disruption of tertiary structure, thereby exposing PPO to a more polar environment. CD spectra showing decreased α-helix and β-turn contents and increased β-sheet and random coil contents confirmed the rearrangement and denaturation of the secondary structure with increased temperature. In conclusion, this study explains the enzymatic browning mechanism by PPO and relates structural modification to PPO activity. A relatively low temperature resulted in inactivation of PPO using the HPCD method, making it a potential technology for the production of high quality fresh juice in which high-temperature should be avoided.

## Figures and Tables

**Table 1:** pH and brix of quince juice from thermal and HPCD treatments.

Treatment	Temp	pH	°Brix
Control (Untreated)		4.74 ± 0.23a	11.55 ± 0.22a
Thermal-treated (20 min)	25	4.75 ± 0.18a	11.54 ± 0.18a
	35	4.73 ± 0.24a	11.56 ± 0.2a
	45	4.73 ± 0.13a	11.54 ± 0.2a
	55	4.70 ± 0.22a	11.51 ± 0.19a
	65	4.69 ± 0.17a	11.49 ± 0.19a
HPCD-treated (20 MPa, 20 min)	25	4.23 ± 0.21ab	11.25 ± 0.37a
	35	4.23 ± 0.23ab	11.19 ± 0.16a
	45	4.20 ± 0.19ab	11.10 ± 0.15a
	55	4.18 ± 0.14ab	10.98 ± 0.17a
	65	4.11 ± 0.11b	10.92 ± 0.21a

Data presented as the mean ± SD (standard deviation). Different letters represent significant difference among means (*p* ≤ 0.05).

**Table 2:** Secondary structure contents of thermal- and HPCD-treated PPO.

Treatments	Secondary Structure Contents					
	α-Helix (%)	β-Sheet (%)	β-Turn (%)	Random Coil (%)	PPO Activity (%)	Concentration (%)
Native	39.34 ± 1.42a	17.9 ± 1.13d	26.41 ± 0.59a	16.35 ± 0.75d	24.23 ± 0.76d	8.57 ± 0.67b
25 °C thermal	38.96 ± 0.96b	18.4 ± 0.78cd	24.48 ± 0.82ab	18.16 ± 1.12cd	42.58 ± 1.02a	12.78 ± 0.93a
25 °C HPCD	35.26 ± 0.78b	20.5 ± 0.53b	23.85 ± 0.77b	20.39 ± 0.82c	36.88 ± 0.64b	4.04 ± 0.15c
65 °C thermal	30.81 ± 1.77c	23.4 ± 0.35b	21.21 ± 1.11c	24.58 ± 0.66b	29.53 ± 1.2c	3.91 ± 0.09c
65 °C HPCD	24.11 ± 0.97d	27.7 ± 0.77a	18.4 ± 1.15d	29.79 ± 0.92a	07.40 ± 1.33e	0.27 ± 0.015d

Data presented as the mean ± SD (standard deviation). Different letters represent significant difference among means (*p* ≤ 0.05).
